# Electronic Properties and Mechanical Stability of Multi-Ion-Co-Intercalated Bilayered V_2_O_5_

**DOI:** 10.3390/ma17133364

**Published:** 2024-07-08

**Authors:** Chunhui Ma, Bo Zhou

**Affiliations:** Institute of Modern Physics, Shaanxi Key Laboratory for Theoretical Physics Frontiers, Northwest University, Xi’an 710127, China

**Keywords:** bilayer V_2_O_5_, first-principles study, electrode materials

## Abstract

Incorporating metal cations into V_2_O_5_ has been proven to be an effective method for solving the poor long-term cycling performance of vanadium-based oxides as electrodes for mono- or multivalent aqueous rechargeable batteries. This is due to the existence of a bilayer structure with a large interlayer space in the V_2_O_5_ electrode and to the fact that the intercalated ions act as pillars to support the layered structure and facilitate the diffusion of charged carriers. However, a fundamental understanding of the mechanical stability of multi-ion-co-intercalated bilayered V_2_O_5_ is still lacking. In this paper, a variety of pillared vanadium pentoxides with two types of co-intercalated ions were studied. The root-mean-square deviation of the V-O bonds and the elastic constants calculated by density functional theory were used as references to evaluate the stability of the intercalated compounds. The d-band center and electronic band structures are also discussed. Our theoretical results show that the structural characteristics and stability of the system are quite strongly influenced by the intercalating strategy.

## 1. Introduction

Lithium-ion batteries (LIBs) are extensively used in the market because of their superior high-energy density [1,2]. However, their future growth has been limited by several key issues, such as resource issues and security. In recent years, aqueous rechargeable batteries (ARBs) with alkaline cations (Li^+^, Na^+^, and K^+^) [3,4,5,6,7] and multivalent cations (Zn^2+^, Mg^2+^, and Al^3+^) [8,9,10] have attracted increasing interest and are considered powerful alternatives to LIBs. There are numerous reports on cathode materials, such as vanadium oxides [11,12,13,14,15], manganese-based materials [16], and Prussian blue [17,18]. Among them, vanadium oxides have drawn attention owing to their flexible multivalent state, remarkable specific capacity, and abundant reserves in nature.

V_2_O_5_ is one of the few electrode materials that are able to accommodate both monovalent and multivalent cations within a layered structure. The layers are connected by van der Waals forces and allow for the (de)intercalation of various ions. Aqueous electrolytes and hydrated vanadium oxide (VOH) electrodes are used in aqueous V_2_O_5_ batteries. The presence of H_2_O modifies the α-V_2_O_5_ structure into a bilayer structure_,_ which has a larger interlayer space, thus facilitating the diffusion of metal ions [14]. The bilayer structure is typical in xerogels, nanosheets, and nanotubes. It was further discovered that the insertion of metal ions can improve the electrode performance by flexibly adjusting the layer spacing and reinforce the stability of VOH by supporting the V–O layer, with the ions acting like pillars. Investigators explored the regulation of the VOH structure after various metal ions were inserted between the VOH layers, such as Na^+^, Ca^2+^, Mg^2+^, and Al^3+^ [12,19,20,21]. Recent studies have found that the organic molecule polyaniline (PANI) can also be inserted between the layers of VOH, resulting in an increase in the layer spacing to 14.08 Å and maintaining the stability of the layered structure [22]. In recent years, the pre-intercalation strategy has also been increasingly applied in zinc batteries. For example, by intercalating polyaniline and water in V_2_O_5_ nanowires, significant capacity gains and improved cycle stability were achieved [23]. Zhang et al. constructed a vanadium oxide hydrate with an tunable P-band center for zinc batteries through a pre-intercalation strategy [24]. Li et al. proposed a 3D flower-like NH_4_V_4_O_10_ micro-morphology with preinserted Mg^2+^ ions as a cathode material for AZIBs, exhibiting a high specific capacity, a satisfactory rate performance, and an excellent life cycle [25]. Although these methods can effectively improve the electrochemical performance of vanadium oxide, metal ions are small in volume and have limited ability to expand layers. Alternatively, co-intercalating, i.e., inserting two or more kinds of extrinsic atoms simultaneously, is considered an effective method to improve the interlayer space and performance of the electrode. Until now, many metal ion pre-intercalation strategies have focused on V_2_O_5_-based cathodes [11,12,13,14,15]. In both single-ion and multiple-ion pre-intercalation, the same fundamental structural unit of the V_2_O_5_ bilayer supported by two different types of ions is used and plays an essential role in determining the structural stability and electrochemical performance of the electrodes. However, there has been no specialized theoretical study focusing on this structural pattern. The different roles of alkali and transition metal cations in improving the stability and the electrochemical performance of vanadium-based materials are still unclear. The advantages and disadvantages of the pillars have not been identified. It is meaningful to fully investigate the effect of co-intercalating pairs composed of common alkali metals and transition metal ions.

This paper focuses on co-pre-inserted combinations of monovalent and divalent/trivalent cations in bilayered V_2_O_5_ as cathode materials for multivalent ion batteries. We mainly discuss the structural stability and electronic properties of bilayered V_2_O_5_ after metal ion intercalation. Our results may open new opportunities for the development of high-performance vanadium oxide-based cathodes for multivalent-cation batteries.

## 2. Methodology

### Computational Details

All the calculations in this work were performed using density functional theory (DFT) with the Vienna Ab Initio Simulation Package (VASP5.4.4) [26,27]. The projector augmented-wave (PAW) method was employed, which has been widely used for battery materials and has shown good predictive capability [28,29,30]. Generalized gradient approximation (GGA) [31] with Perdew–Burke–Ernzerhof (PBE) parameterization was applied as the exchange-correlation functional. Although Hubbard U correction is usually employed since it accounts for on-site Coulomb interactions for the 3d orbitals of V, it was not included due to the sufficiency of PBE parameterization for voltage calculations and the poor convergence previously observed with the +U correction for diffusion calculations [32]. All calculation parameters were selected after the convergence test of the total energy of the system. The cutoff energy of the plane wave was set to 500 eV. The Brillouin zone of the unit cells was sampled by a 4 × 4 × 4 mesh according to the Monkhorst–Pack scheme. The energy and the force convergence criterion were chosen to be 10^−7^ eV and 10^−3^ eV Å^−1^, respectively.

In order to investigate the thermodynamic stability of the structure after intercalation, the formation energy was calculated. The formation energy is defined as follows:

$$E_{formation}=E_{ABV_{8}O_{20}}-E_{V_{8}O_{20}}-E_{A}-E_{B}$$

where $E_{ABV_{8}O_{20}}$ is the total energy of the co-intercalated system, $E_{V_{8}O_{20}}$ is the energy of the bilayer V_2_O_5_ crystal, and $E_{A}$ and $E_{B}$ are the energy per atom of metal ions A/B, respectively, obtained from bulk metallic calculations. With this definition, a negative $E_{formation}$ means that the intercalating process is exothermic, and a large absolute value means increased stability.

**Figure 1:**
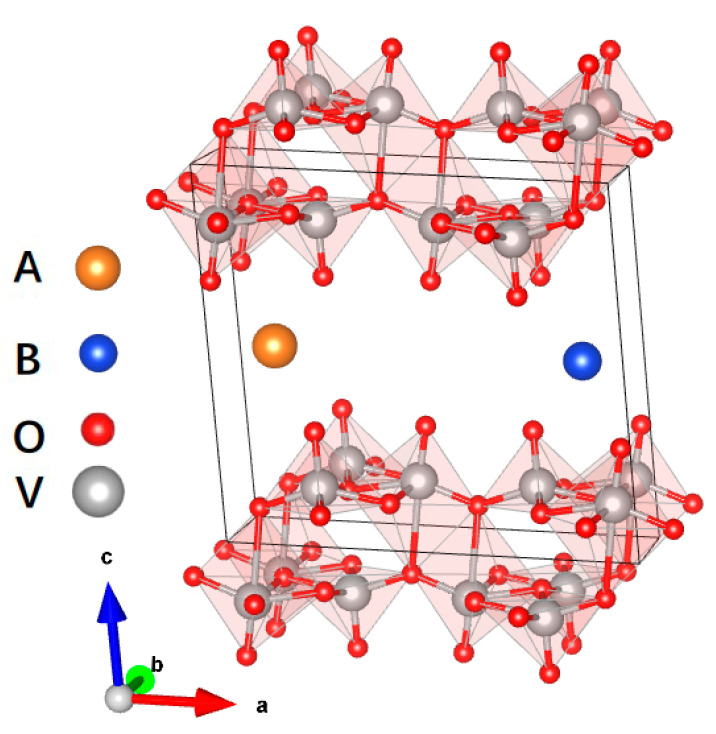
The structure of AB-V_8_O_20_. The orange sphere represents the intercalated atom A, the blue sphere represents the intercalated atom B, and gray and red spheres represent vanadium and oxygen atoms, respectively.

In addition to the thermodynamic stability, the mechanical stability of the intercalated crystals was studied. To judge the stability of the intercalated crystals, elastic stability conditions were adopted [33,34]. For the crystal axes XX-1, YY-2, ZZ-3, XY(YX)-4, YZ(ZY)-5, and XZ(ZX)-6, Voigt representation was adopted, and the axis arrangement is shown in Figure 1. Bilayer V_2_O_5_ belongs to the monoclinic crystal system. When two cations are introduced, its crystal system remains within the monoclinic category. There are 13 independent elastic constants associated with it, and the elastic constant matrix is diagonal. The stability criteria of the monoclinic crystal system are as follows [35,36]:

$$C_{11}>0,\;C_{22}>0,\;C_{33}>0,\;C_{44}>0,\;C_{55}>0,\;C_{66}>0$$

$$(C_{22}+C_{33}-2C_{23})>0,\;[C_{11}+C_{22}+C_{33}+2(C_{12}+C_{13}+C_{23})]>0$$

$$(C_{33}C_{55}-C_{35}^{2})>0,\;(C_{44}C_{66}-C_{46}^{2})>0$$

$$[C_{22}(C_{33}C_{55}-C_{35}^{2})+2C_{23}C_{25}C_{35}-C_{23}^{2}C_{55}-C_{25}^{2}C_{33}]>0$$

$$g=C_{11}C_{22}C_{33}-C_{11}C_{23}^{2}-C_{22}C_{13}^{2}-C_{33}C_{12}^{2}+2C_{12}C_{13}C_{23}$$

$$\begin{array}{l} \left[2C_{15}C_{25}(C_{33}C_{12}-C_{13}C_{23})+C_{15}C_{35}(C_{22}C_{13}-C_{12}C_{23}\right)\\ +C_{25}C_{35}(C_{11}C_{23}-C_{12}C_{13})-C_{15}^{2}(C_{22}C_{33}-C_{23}^{2})\\ +C_{25}^{2}(C_{11}C_{33}-C_{13}^{2})+C_{35}^{2}(C_{11}C_{22}-C_{12}^{2})+C_{55}g]>0 \end{array}$$

## 3. Results and Discussion

### 3.1. Structure Modification after Co-Intercalation

The structural instability of the common orthorhombic α-V_2_O_5_ (space group Pmmn) phase has emerged as a constraint for multivalent-cation battery design. The interplanar spacing between the [VO_5_−VO_5_] polyhedral chains in α-V_2_O_5_ may not be sufficient to reversibly accommodate large-ionic-radius cations within its crystal volume. Meanwhile, the presence of large 2D channels for ion transport and the very thin solid network of V_2_O_5_·nH_2_O-derived materials stimulate further investigation regarding their potential application as electrode materials in multivalent cation batteries. X-ray diffraction (XRD) measurements revealed that V_2_O_5_·nH_2_O xerogels are composed of bilayers of V_2_O_5_ (monoclinic, space Group C2/m) separated by water layers [37]. The bilayer V_2_O_5_ comprises two layers, each with the V_2_O_5_ stoichiometry facing each other, which confers distinct electrochemical behavior to the V_2_O_5_ gel. Special focus is being put on incorporating metal cations into bilayer V_2_O_5_ to improve the capacity and cyclic stability and maintain the bilayer structure of the V_2_O_5_ frame [38,39,40,41,42].

Compared to the single-cation intercalation strategy, a co-intercalating strategy with two types of cations could be more attractive. Considering economics and practicality, we chose alkali metals (Li, Na, K), alkaline earth metals (Mg, Ca) and transition metals in the first four cycles (Sc, Ti, V, Cr, Mn, Fe, Co, Ni, Cu, Zn). A 1 × 2 × 1 supercell of bilayer V_2_O_5_ was used, and we fixed the intercalation positions of intercalation atoms A and B at two symmetric positions, (0.125, 0.5, 0.5) and (0.875, 0.5, 0.5), as suggested for ν-V_2_O_5_ and δ-V_2_O_5_, which are analogous to bilayer V_2_O_5_ [43]. The cell parameters and atomic positions were further optimized by means of DFT calculations, as shown in Figure 1. One A and one B ion were intercalated into the bilayer V_2_O_5_, giving a stoichiometric formula of ABV_8_O_20_. Our theoretical model is reasonable considering the pre-intercalated metal ions in bilayer V_2_O_5_ that have previously been investigated, such as NH_4_V_4_O_10_ [44], Mg_0.34_V_2_O_5_ [12], and Na_0.33_V_2_O_5_ [19].

Water molecules are essential in the electrochemical process of aqueous rechargeable batteries [45]. Structural water, a common pre-intercalated molecule, can expand the layer spacing and effectively shield the charge interaction of metal ions. In published works, water molecules have been shown to have a positive effect on the stability of the electrode. This is not the main factor in the instability of the electrode. The hydrated cation and proton dynamics are complicated and are beyond the scope of this manuscript.

**Figure 2:**
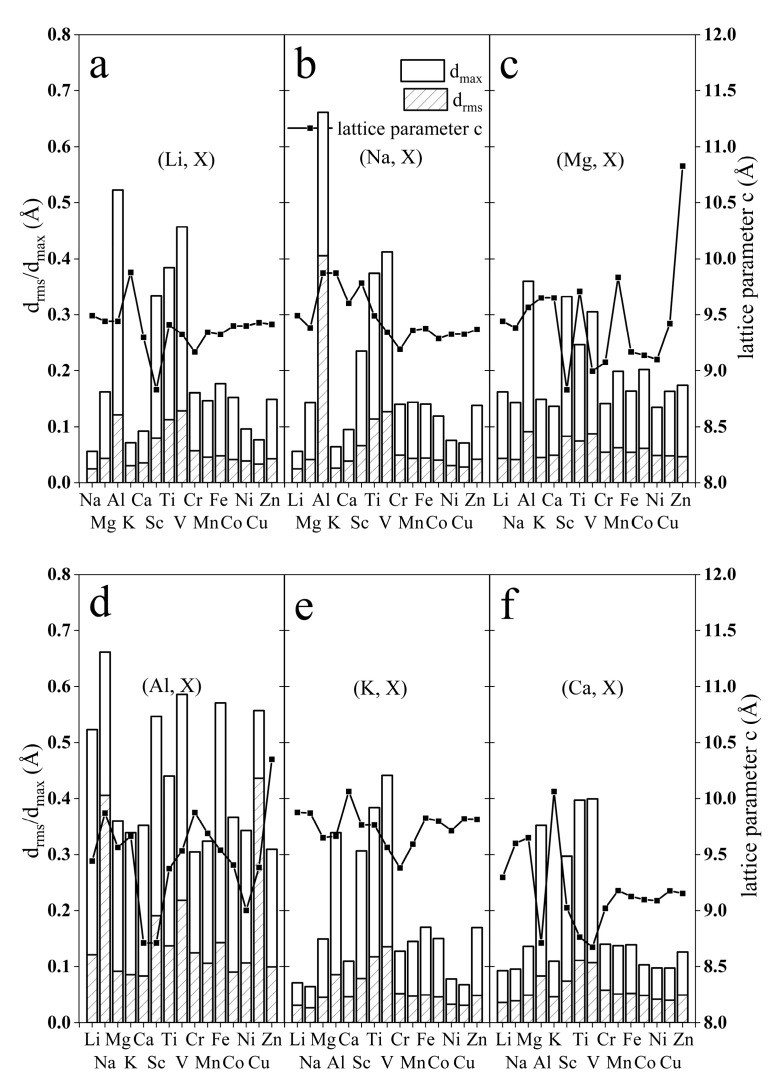
Structural changes under different intercalation combinations. (**a**–**f**) Structural changes in Li-, Na-, Mg-, Al-, K-, and Ca-dominated combinations. The bar chart shows the change in bond length, and the broken line chart shows the change in layer spacing.

The optimized unit cell parameters for bilayer V_2_O_5_ are a = 11.69 Å, b = 3.61Å, c = 9.85 Å, α = 90°, β = 96.47°, and γ = 90°, which are in reasonable agreement with the experimental values reported in Ref. [43]. The mean deviation is about 1.5%. The optimized lattice parameters of pure bilayered V_2_O_5_ obtained using different methods are shown in Table 1 [46]. With the introduction of metal ions, the layer spacing of V_2_O_5_ decreases due to the strong ionic bonds with the vanadium oxide bronze. As the structures both before and after intercalation exhibit clear layering, the c-axis lattice constant was chosen in this paper as an indicator of layer spacing. As illustrated in Figure 2, the incorporation of Mg (ionic radius 72 pm) [47], Ca (100 pm), or Sc (75 pm) ions may decrease the layer spacing due to their strong correlation with the V-O framework, while Al (53 pm) and K (151 pm) may enlarge the layer spacing, accompanied with significant structural changes.

In order to gain further insights into the intercalated structure and estimate the deviation of the co-intercalated structure from the pristine one, the root-mean-square deviation (d_rms_) of the 32 V-O bonds involved in our model was calculated. The maximum deviation of the V-O bond lengths (d_max_) is also shown in Figure 2. The apex of the square pyramid is a vanadyl bond (V-O) that is shorter (1.5–1.6 Å) than the other V-O bonds (1.8–2.1 Å). The root-mean-square deviation of the 32 V-O bonds serves as a reference value to evaluate the structural stability after ion intercalation. We took 0.1 Å as the lower limit of d_rms_, which suggests potential damage to the layered structure. This standard is not mandatory.

For the Li-doped cases, as shown in Figure 2a, the combinations of (Li, Na), (Li, K), (Li, Ca), and (Li, Ni) exhibited slight deviations from pristine bilayered V_2_O_5_, which indicates stability with pillars supporting the bilayer structure. For the Na-doped cases, the (Na, Li), (Na, K), and (Na, Ni) pairs exhibited a small deviation, which can be judged from the d_rms_ and d_max_ values. Additionally, co-intercalations of (K, Ni), (K, Cu), (Ca, K), (Ca, Co), (Ca, Ni), and (Ca, Cu) showed a minimal influence on the bilayer structure.

Although the ionic size of Al^3+^ (0.53Å) [47] is smaller than that of Li^+^ (0.76 Å), the high charge density of trivalent Al^3+^ cations can lead to strong interactions between the Al^3+^ cations and the framework of the main structure, potentially resulting in structural changes or slow kinetics during the process of Al^3+^ intercalation. The incorporation of aluminum ions dramatically changes the bilayer structure in each case. Also, the d_rms_ value is relatively large, more than 0.1 Å. With the involvement of Al^3+^, the bilayer V_2_O_5_ suffers from structural phase transformation. Our theoretical results indicate a similar trend for cases involving Sc and Ti ions.

Bilayer V_2_O_5_ is widely used as a cathode for aqueous zinc-ion batteries (ZIBs). Mai’s group reported Na_0.33_V_2_O_5_ (NVO) with enhanced performance (253.7 mA h g^−1^ @ 200 mAg^−1^) [19]. Husam N. Alshareef’s group also demonstrated that a better ZIB performance could be achieved on Ca_0.25_V_2_O_5_·nH_2_O (CVO) (340 mA h g^−1^ @ 50 mAg^−1^) [20] and Mg_0.34_V_2_O_5_·nH_2_O (340 mA h g^−1^ @ 100 mA g^−1^) [12]. Liang’s group further developed several kinds of potassium vanadate nanobelts (K_2_V_8_O_21_, K_0.25_V_2_O_5_, K_2_V_6_O_16_·1.57H_2_O, and KV_3_O_8_) [49] for ZIBs. The root-mean-square deviation (d_rmx_) values for (Na, Zn), (Ca, Zn), (Mg, Zn), and (K, Zn) are 0.041, 0.048, 0.047, and 0.047 Å, which are smaller than the 0.1 Å standard for stability analysis. Shuhua Wang’s group reported a reversible Zn//(Na, Mn)V_8_O_20_·nH_2_O system (377 mA h g^−1^@ 100 mA g^−1^), achieved by introducing manganese (Mn) ions into NaV_8_O_20_ [8]. In Wang’s work, the c-axis lattice constant of (Na_0.43_Mn_0.53_)V_8_O_20_·nH_2_O was 10.62 Å, while that of (Na, Mn)V_8_O_20_ obtained by our calculations was 9.36 Å. We attribute this difference to the fact that our system does not consider the unique role of water in expanding the layer spacing. A d_rms_ value of 0.043 Å was obtained for the (Na, Mn) system, indicating that this co-intercalating strategy can maintain the bilayer structure and facilitate the diffusion of Zn ions. Our stability analysis based on the root-mean-square deviation of V-O bonds is consistent with these reported metal-ion-pre-intercalated V_2_O_5_ experiments.

### 3.2. Stability and Mechanical Properties after Co-Intercalation

**Figure 3:**
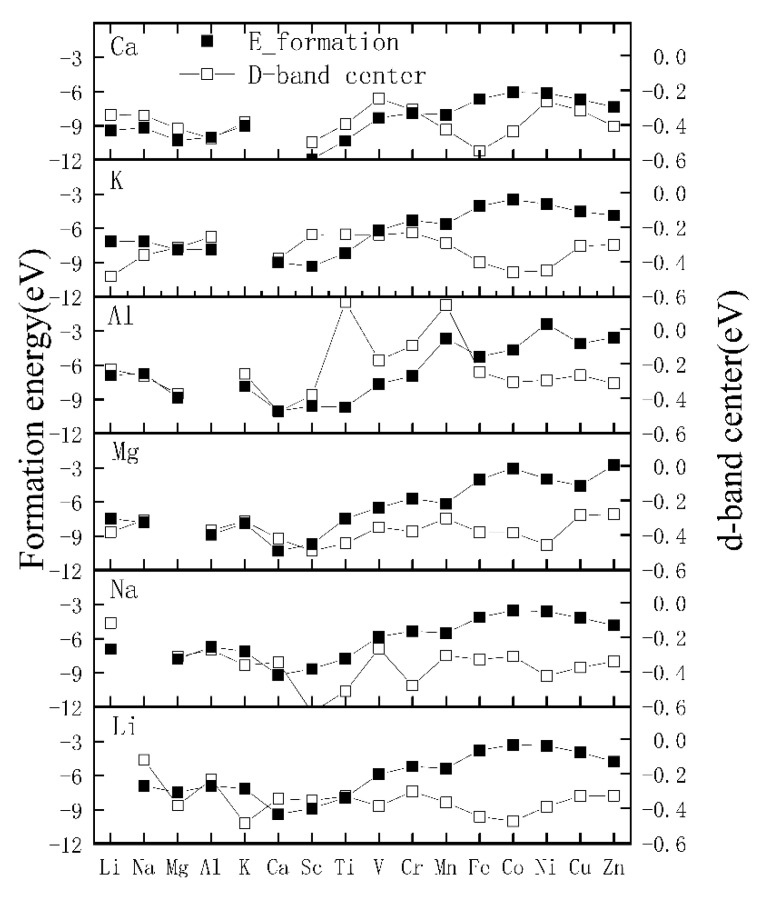
Formation energy and d-band center of different intercalation combinations.

To provide further insight into the stability of the bilayer V_2_O_5_ after cation intercalation, we also calculated the formation energies, shown in Figure 3. All the co-intercalated systems have negative formation energy, indicating that they are thermodynamically stable and can easily be synthesized by sol–gel chemistry or hydrothermal methods.

To further estimate the mechanical stability of V_2_O_5_ bilayers with various guests, the elastic constants of these materials were calculated. The diagonal elastic constants C_ii_ (C_11_, C_22_, C_33_) are dominant and are listed in Table 2. Based on these elastic constants, there are 62 stable pairs and 13 unstable pairs among the 75 co-intercalated pairs considered. The unstable co-intercalated systems that did not meet the stability criterion, as outlined in the methodology section, were marked as “*”.

The combinations (Li, Al), (Na, Al), (Al, Sc), (Al, Cu), and (Al, Zn)-V_8_O_20_ involving Al^3+^ are unstable, which is consistent with the aforementioned V-O bond deviation analysis. This may be related to the serious structural deformation of (Al, B)-V_8_O_20_, which leads to damage to the bilayer structure. We noticed a recent work by Q. Pang et al. that presented aluminum-pre-intercalated V_2_O_5_ as a high-performance cathode material for aqueous zinc-ion batteries [21]. Their powder X-ray diffraction (XRD) study showed an orthorhombic V_2_O_5_ structure, while no monoclinic bilayer structure was detected. In a recent work by P. De et al., two-dimensional V_2_O_5_ nanosheets were reported as a cathode material for realizing low-cost aqueous aluminum-ion batteries. The formation of layered orthorhombic V_2_O_5_ with the Pmmn space group was also confirmed by XRD diffraction [50].

The mechanical properties of an electrode are also an essential consideration in the design of mono- and multivalent cation batteries. Assuming that electrode particles are typically polycrystalline and can be modeled as isotropic elastic materials, the averaged bulk (B), shear (G), and Young’s (E) moduli and Poisson’s ratio (ν) can be obtained from the C_ij_ values based on Reuss’s lower bound, Voigt’s upper bound, and Hill’s homogenization schemes [51]. These values were calculated with the help of the software VASPKIT (1.5.0) [52]. The calculated results are shown in Table 2, and the Voigt approximation was still adopted. The bulk modulus, Young’s modulus, and shear modulus of the pristine bilayer V_2_O_5_ were 64.09, 69.43, and 26.31 GPa. The data in Table 2 suggest that the stiffness of the material is enforced by co-intercalation.

According to the criterion of fracture behavior, materials with a low Poisson’s ratio are brittle materials [53]. A Poisson’s ratio of ν < 0.26 indicates that the material is brittle, while ν > 0.26 indicates that the material is ductile [54]. Ductile materials are expected to be used as electrode materials for flexible batteries. The elastic constant C_33_ relates to interlayer interactions, which also have a strong influence on the flexibility of the electrode. The smallest C_33_ was observed for (Mg, Ti) co-intercalation, while the largest value was from the (Al, Cr) case, indicating a denser structure.

The electrode materials can be stiffened by Ca intercalation, as judged from the elastic constants. More attention should be paid to K ions, since the formation energy is negative, the structural deviation after the intercalation is relatively small, the interlayer spacing is overall large compared with the other cases, and the d-band center indicates lesser H_2_ generation with all cases involving K. K ions seem to be good candidates as pillars for bilayer V_2_O_5_.

Due to the rich structural chemistry of vanadium oxide frameworks, our work gives ideas of interest for research on V_2_O_5_-based electrode materials for battery applications. However, some points should be further investigated, such as the interactions between the intercalating sites and the role of structural water in assisting cation insertion. Continued efforts are underway to solve these problems. A validated “cocktail method” for vanadium-based electrode material design may appear after further studies.

### 3.3. Electronic Structure

Density functional theory (DFT) calculations were carried out to study the electronic band structure of vanadium oxide before and after guest intercalation. The main characteristic of the band structure of V_2_O_5_-based material is the existence of split bands separated from higher conduction bands, which are weakly dispersing due to V d_xy_ orbitals overlapping with O 2p. Due to the intercalation of alkali ions, the split-off bands of most of the intercalation configurations are partially occupied, leading to charge ordering and interesting 1D magnetic properties. The intercalated V_2_O_5_ electrodes show good electrical conductivity overall. We also noticed that the (Mg, Ca), (Mg, Sc), (Mg, Cr), (Ca, Cr), (Ca, Mn), and (Ca, Zn)-V_8_O_20_ systems are obvious semiconductors with a small band gap, which indicates poor electrical conductance, as shown in Table 2.

Water splitting in aqueous environments should be considered when designing materials for advanced ARBs [55]. Water decomposition will not only cause electrolyte consumption, resulting in gas expansion of the battery and safety problems, but also consume electrons and reduce the coulomb efficiency of the battery [45]. This process involves two half-reactions of the oxygen evolution reaction (OER) and hydrogen evolution reaction (HER), which exhibit different reaction mechanisms in materials with different d-band centers. The d-band center is an effective indicator of hydrogen solution and diffusion [56]. A shift upward (downward) of the d-band center enhances (weakens) hydrogen solution ability. The d-band centers of the (Al, Mn), (Al, Ti)-V_8_O_20_ pairs are positioned above the Fermi energy, which warrants further attention. The (Li, Na), (Al, Cr), and (Al, V) pairs tend to boost hydrogen generation, while the (Li, K), (Na, Sc), and (Ca, Sc) pairs suppress such generation, as illustrated in Figure 3.

## 4. Conclusions

Two-dimensional bilayer V_2_O_5,_ which offers the advantage of a more open framework for ion intercalation, is an attractive cathode material for aqueous rechargeable batteries. Multi-ion co-pre-intercalation is an effective method used to modify electrodes, stabilize layered structures, and improve electrochemical performance. Co-intercalation cases involving two types of ions were studied systematically. This theoretical study mainly focused on the changes after ion intercalation in terms of the bond length, electronic structure, stability, and mechanical properties. The calculation results showed that the (Li, Al), (Na, Al), (Na, Sc), (Na, Co), (Na, Ni), (Mg, Ti), (Mg, Fe), (Al, Sc), (Al, Cu). (Al, Zn), (K, Ca), (Ca, Co), and (Ca, Ni) pairs are not stable. In contrast, the (Li, Na), (Na, K), (Li, K), and (K, Cu) pairs presented a small deviation from the pristine layered structure. As judged from the root-mean-square deviation of the V-O bonds, the K ion can enlarge the interlayer space and maintain the layer structure. Incorporating Al, Ti, Sc, and V harms the bilayer structure. Calculations of the d-band center revealed that the (Li, K), (Na, Sc), and (Ca, Sc) pairs can suppress water splitting on the surface of the electrode. Our work provides more co-intercalation schemes and insights into vanadium oxide-based electrode materials.

## Figures and Tables

**Table 1:** Calculated lattice parameters of pure V_2_O_5_ obtained using different methods.

D-V_2_O_5_	*a* (Å)	*b* (Å)	*c* (Å)
PBE (this work)	11.69	3.61	9.85
DFT-PBE-D3 [48]	11.36	3.56	9.37
PBE + U [46]	11.58	3.65	8.59

**Table 2:** Elastic constants and elastic modulus for different intercalation situations. The Voigt notation replaces XX→1, YY→2, ZZ→3, ZY(YZ)→4, XZ(ZX)→5, and XY(YX)→6. All values are in units of GPa.

	C_11_	C_22_	C_33_	B (GPa)	E (GPa)	G (GPa)	v	
Li, Na	175.26	220.71	41.20	86.30	84.67	31.68	0.34	metal
Li, Mg	176.67	223.58	39.78	92.33	75.35	27.62	0.36	metal
Li, Al *	137.08	229.34	44.59	72.64	63.91	23.61	0.35	metal
Li, K	167.36	214.76	34.34	81.77	86.38	32.62	0.32	metal
Li, Ca	191.37	242.23	43.84	99.24	96.45	36.04	0.34	metal
LI, Sc	172.38	225.90	89.33	99.16	90.72	33.66	0.35	metal
Li, Ti	107.21	235.99	96.20	89.31	82.64	30.71	0.35	metal
Li, V	117.16	240.22	100.75	91.08	88.82	33.20	0.34	metal
Li, Cr	181.39	238.00	73.39	102.71	97.23	36.22	0.34	metal
Li, Mn	181.03	242.48	64.85	100.64	90.84	33.65	0.35	metal
Li, Fe	174.04	230.18	48.86	95.77	76.12	27.83	0.37	metal
Li, Co	175.50	227.10	27.49	84.06	82.85	31.01	0.34	metal
Li, Ni	180.49	225.17	35.08	92.08	88.26	32.93	0.34	metal
Li, Cu	174.10	229.84	39.47	89.79	84.08	31.28	0.34	metal
Li, Zn	183.26	227.57	45.33	92.43	86.73	32.28	0.34	metal
Na, Mg	192.11	229.62	52.22	95.88	94.63	35.43	0.34	metal
Na, Al *	134.19	213.54	27.69	67.29	70.94	26.78	0.32	metal
Na, K	163.82	210.07	35.39	80.41	84.65	31.96	0.32	metal
Na, Ca	176.04	236.56	45.93	93.15	92.19	34.53	0.34	metal
Na, Sc *	168.74	227.96	42.56	86.82	76.12	28.11	0.35	metal
Na, Ti	132.90	236.19	64.50	85.31	83.13	31.07	0.34	metal
Na, V	139.34	224.61	81.76	81.30	91.10	34.69	0.31	metal
Na, Cr	188.66	231.72	67.35	99.89	98.81	37.01	0.34	metal
Na, Mn	192.04	228.80	51.41	95.43	94.42	35.36	0.34	metal
Na, Fe	184.22	220.59	41.70	95.18	86.56	32.10	0.35	metal
Na, Co *	182.06	235.02	27.00	78.03	81.14	30.58	0.33	metal
Na, Ni *	171.86	229.76	62.91	79.50	90.02	34.32	0.31	metal
Na, Cu	173.65	223.93	47.30	89.93	80.87	29.95	0.35	metal
Na, Zn	191.81	225.46	48.41	95.09	93.25	34.89	0.34	metal
Mg, Al	181.36	257.38	62.95	99.18	103.06	38.84	0.33	metal
Mg, K	179.63	225.05	39.73	90.08	100.97	38.45	0.31	metal
Mg, Ca	207.43	248.33	68.19	108.05	111.40	41.94	0.33	0.16
Mg, Sc	190.59	202.86	101.40	93.06	95.58	35.97	0.33	0.19
Mg, Ti *	178.62	224.56	16.78	84.77	69.08	25.32	0.36	metal
Mg, V	215.85	255.24	89.67	116.66	100.84	37.20	0.36	metal
Mg, Cr	216.94	252.68	63.09	113.40	97.13	35.78	0.36	0.15
Mg, Mn	175.28	252.18	59.21	99.06	90.10	33.41	0.35	metal
Mg, Fe *	192.59	240.38	39.68	101.32	70.01	25.28	0.38	metal
Mg, Co	155.68	216.09	36.41	72.94	77.57	29.32	0.32	metal
Mg, Ni	178.05	243.94	63.53	101.67	88.47	32.65	0.35	metal
Mg, Cu	176.35	228.65	47.06	95.33	81.21	29.90	0.36	metal
Mg, Zn	138.83	181.10	25.19	63.24	68.06	25.77	0.32	metal
Al, K	144.54	226.89	30.74	74.99	94.67	36.71	0.29	metal
Al, Ca	190.85	248.94	110.96	113.22	112.94	42.34	0.33	metal
Al, Sc *	177.47	220.54	86.65	95.23	77.35	28.34	0.36	metal
Al, Ti	190.12	278.23	75.31	102.18	122.25	47.00	0.30	metal
Al, V	174.82	265.06	63.13	99.00	112.96	43.12	0.31	metal
Al, Cr	203.13	305.86	144.41	133.42	155.83	59.69	0.31	metal
Al, Mn	140.36	219.45	43.54	71.93	78.79	29.90	0.32	metal
Al, Fe	169.46	249.93	56.82	98.69	97.10	36.34	0.34	metal
Al, Co	182.68	261.93	66.51	101.06	108.13	40.91	0.32	metal
Al, Ni	154.21	253.78	43.35	83.72	85.90	32.32	0.33	metal
Al, Cu *	143.62	219.17	31.41	70.33	53.84	19.61	0.37	metal
Al, Zn *	118.89	204.58	33.62	66.12	66.29	24.87	0.33	metal
K, Ca *	156.52	219.61	54.43	87.04	62.61	22.68	0.38	metal
K, Sc	154.01	223.96	46.59	81.08	85.07	32.10	0.33	metal
K, Ti	116.71	221.52	89.44	77.60	94.77	36.55	0.30	metal
K, V	155.35	231.92	101.99	92.75	104.50	39.82	0.31	metal
K, Cr	186.73	227.42	54.76	96.52	106.68	40.54	0.32	metal
K, Mn	187.15	223.47	45.57	92.77	101.68	38.59	0.32	metal
K, Fe	166.40	209.33	34.01	81.24	87.45	33.11	0.32	metal
K, Co	164.14	220.29	40.81	83.72	91.57	34.75	0.32	metal
K, Ni	167.36	217.93	37.09	86.53	87.23	32.74	0.33	metal
K, Cu	176.80	225.94	38.47	89.41	84.04	31.28	0.34	metal
K, Zn	170.14	218.06	36.94	83.59	89.45	33.84	0.32	metal
Ca, Sc	190.92	249.69	66.62	106.57	108.02	40.58	0.33	metal
Ca, Ti	191.04	240.23	132.97	112.17	109.79	41.06	0.34	metal
Ca, V	194.87	244.95	130.29	114.00	116.08	43.63	0.33	metal
Ca, Cr	201.92	251.90	94.50	113.01	117.09	44.11	0.33	0.17 †
Ca, Mn	205.68	248.31	66.70	107.45	110.49	41.58	0.33	0.13
Ca, Fe	205.88	252.55	75.81	110.53	111.69	41.94	0.33	metal
Ca, Co *	178.11	251.16	68.70	103.27	105.75	39.78	0.33	metal
Ca, Ni *	183.65	244.71	83.99	105.95	90.26	33.23	0.36	metal
Ca, Cu	177.89	246.19	68.00	99.96	94.01	34.99	0.34	metal
Ca, Zn	208.13	247.74	70.06	108.41	110.38	41.49	0.33	0.19

* represents the unstable pairs; † represents the direct band gap.

## Data Availability

The raw data supporting the conclusions of this article will be made available by the authors on request.
